# Motion mitigation in positron-emission tomography guided radiotherapy delivered on a magnetic resonance imaging-linear accelerator^☆^

**DOI:** 10.1016/j.phro.2026.100970

**Published:** 2026-04-17

**Authors:** Albert J. Everard, Rodrigo José Santo, Pim T.S. Borman, Prescilla Uijtewaal, Martin F. Fast, Hugo W.A.M. de Jong, Cornelis A.T van den Berg, Bas W. Raaymakers, Casper Beijst

**Affiliations:** a Department of Radiotherapy, University Medical Center Utrecht, Heidelberglaan 100 3584 CX Utrecht, the Netherlands; b Department of Radiology, University Medical Center Utrecht, Heidelberglaan 100 3584 CX Utrecht, the Netherlands; c Computational Imaging Group, University Medical Center Utrecht, Heidelberglaan 100 3584 CX Utrecht, the Netherlands

**Keywords:** PET-guided radiotherapy, MR-linac, PET/CT, MLC-tracking, Motion Mitigation

## Abstract

**Background and Purpose:**

The precision of Positron Emission Tomography (PET)-guided radiotherapy may be compromised by motion. To address this, we demonstrated the feasibility of a motion-robust PET-to-magnetic resonance linear accelerator (MR-linac) pipeline.

**Materials and Methods:**

For comparison, PET images of a phantom with a target in a movable insert were acquired under a patient-derived breathing waveform and static conditions. Motion correction using the PET Maximum-Likelihood Motion and Activity (MLMA) reconstruction was compared with static reference and motion-blurred images. Targets were delineated using thresholding based on maximum standardized uptake value, and Intensity-Modulated Radiation Therapy (IMRT) plans were generated. Plans were delivered on an MR-linac with and without gating or Multi Leaf Collimator (MLC) tracking. Dosimetry was assessed using film measurements, evaluating dose differences and 3%/3 mm gamma pass rates. The pipeline was further evaluated using clinical PET data, delivered with and without gating and tracking after motion correction. Dosimetric evaluation was performed using a cylindrical multi-diode phantom.

**Results:**

Motion-blurred PET produced larger gross target volumes (GTV) (GTV: 58 cm^3^, GTV_boost_: 14 cm^3^) than motion-corrected PET (GTV: 52 cm^3^, GTV_boost_: 8 cm^3^), the latter matching the static reference (GTV: 52 cm^3^, GTV_boost_: 9 cm^3^). Motion correction reduced dose discrepancies and improved gamma pass rates (72%), with further gains from gating (90.3%) and MLC tracking (98.3%). The measurements of patient simulations showed higher gamma pass rates for the motion-corrected plan (static-delivery: 81.8%, gating: 99.2%, MLC-tracked: 100%) compared to motion-blurred plan (static-delivery: 49.4%, gating: 65.6%, MLC-tracked: 63.6%).

**Conclusions:**

A motion-robust PET-to-MR-linac pipeline using motion-corrected PET imaging and MR-guided motion mitigation is technically feasible and enhances dose precision.

## Highlights

• Motion mitigation raised PET-guided Radiotherapy precision by 51% γ-pass rate.
• GTV decreased 6 cm^3^ and GTV_boost_ 5 cm^3^ due to PET motion correction.
• Solely PET motion correction outperformed delivery correction by 25% γ-pass rate.

## Graphical abstract

**Figure:**
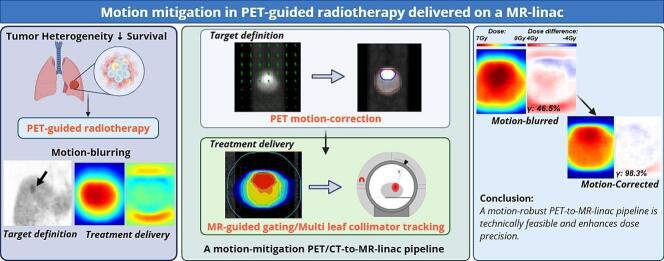

## 1 Introduction

Positron Emission Tomography (PET) provides biological information of oncological disease with sensitivity in the picomolar range. Owing to this sensitivity and the specificity of tumor-targeted radiotracers, PET can provide essential biological information for radiotherapy. The integration of PET-guided radiotherapy aims to leverage this biological information to improve treatment outcomes.

Several novel PET-guided radiotherapy strategies have been proposed, including dose painting, response monitoring and adaptive fractionation based on residual PET signal of the tumor [1], [2], [3], [4]. For example, adaptive radiotherapy based on fluorine-18 fluorodeoxyglucose (FDG)-PET has been reported to be beneficial for overall survival (OS) without an increase in toxicity in non-small cell lung carcinoma (NSCLC) [5]. However, some studies have reported mixed or inconclusive results, largely due to the complexity of these strategies and their reliance on delivering high doses to small volumes [2], [4]. As such, careful assessment of uncertainties in both imaging and dose delivery is warranted [2].

One major source of uncertainty in radiotherapy is motion, for example induced by respiration, patient repositioning and head roll. Motion during simulation imaging introduces blurring artifacts in PET images, leading to an artificially enlarged target volume and reduced standard uptake value (SUV), which can cause systematic errors in radiotherapy planning [6]. During treatment delivery, intra-fraction motion blurs the dose distribution, leading to geometric dose errors that can result in underdosing the target and overdosing organs at risk (OARs) [7], [8].

Therefore, motion mitigating strategies are required in both target definition and treatment delivery. Clinically available methods such as gated PET/computed tomography (CT) and gated delivery can be integrated into the current cone-beam computed tomography (CBCT)-linac workflow, as demonstrated in phantom studies [9]. However, these methods rely on gating, which introduces distinct limitations: gated imaging suffers from reduced statistical quality and is limited to periodic motion, while gated delivery typically results in prolonged treatment times [10], [11].

A novel solution for PET-guided radiotherapy is a dedicated PET-linac system that integrates PET detectors and a linear accelerator on the same rotating gantry, thereby enabling radiation delivery to a moving target [12], [13]. While this approach enables integration of biological information into radiotherapy, it also has limitations. In particular, target tracking relies solely on PET signals, which have low spatial resolution and limited anatomical context, potentially leading to suboptimal tracking and insufficient intra-fraction motion mitigation.

By contrast, the magnetic resonance imaging-linear accelerator (MR-linac) uses MR images (MRI) for target tracking, offering superior resolution and soft-tissue contrast. This enables more accurate monitoring of targets and OAR, resulting in improved motion mitigation during delivery. However, the MR-linac lacks the crucial biological information provided by PET. Incorporating motion-corrected PET/CT images into the MR-linac workflow could therefore combine sensitive biological targeting of cancer cells with MR-guided stereotactic radiotherapy. We therefore propose a motion-mitigation PET/CT-to-MR-linac pipeline to address motion-related inaccuracies during both target definition and treatment delivery [14].

The aim of this phantom study was to demonstrate the feasibility of a motion-robust PET/CT-to-MR-linac workflow in which motion is mitigated during both target definition and treatment delivery, thereby enabling precise PET-guided radiotherapy on a MR-linac.

## 2 Materials and methods

### 2.1 Experimental setup and workflow

**Fig. 1:**
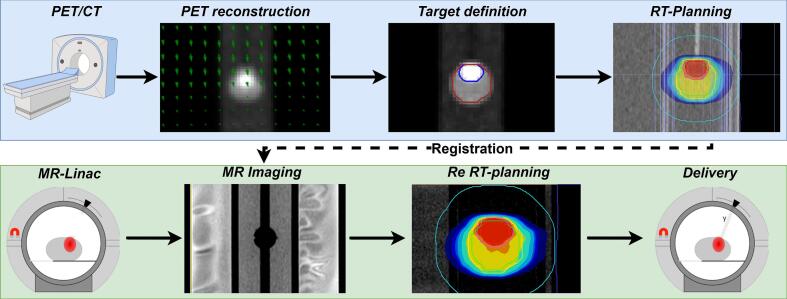
Overview of the PET/CT-to-MR-linac pipeline. The steps related to target definition are indicated in blue and include PET acquisition of raw list-mode data, reconstruction and motion correction, target delineation, and radiotherapy planning. The treatment delivery steps, shown in green, are performed on the MR-linac and consist of MR imaging, adaptation of the radiotherapy plans, and treatment delivery with or without gating and MLC tracking. (For interpretation of the references to colour in this figure legend, the reader is referred to the web version of this article.)

The workflow consisted of two steps: the target definition and the treatment delivery (Fig. 1). The target definition step involved PET/CT acquisition of the raw list mode data, reconstruction and motion estimation and correction of the PET images, target delineation and radiotherapy (RT) planning. The treatment delivery step consisted of MRI acquisition, registration of the PET/CT-based RT plan to the MRI, plan adaptation based on the MRI and subsequent delivery on the MR-linac.

The workflow was tested using the QUASAR™ MRI^4D^ Motion Phantom (IBA Dosimetry GmbH, Schwarzenbruck, Germany). This MR-compatible phantom consists of a fillable body oval (20 cm (L) x 30 cm (W) x 20 cm (H)), with interchangeable inserts. For this experiment, the insert was moved according to a one dimensional (1D) breathing-motion trace extracted from an MRI acquisition on a patient scanned for clinical purpose. The trace had a maximum amplitude of 2 cm, a mean period of 6 s, and no drift.

For the target definition, an insert with a three dimensional (3D)-printed fillable target (5 cm diameter) was used. To mimic a heterogeneous tumor, the target consisted of a porous outer rim and a spherical void (2.5 cm diameter). The pore size (1.8 mm3) was smaller than the scanner’s spatial resolution, resulting in two seemingly homogeneous compartments with a 2:1 ratio for the PET tracer concentration in the void volume with respect to the porous volume, thereby creating a hotspot in the target [14]. The target was filled with 16 kBq/ml F^18^-FDG, achieving an 8:1 spherical void-to-background ratio. For the treatment delivery, an insert was used which could hold a film cassette, into which an EBT3 Grafchromic^TM^ film (Ashlans, Wilmington, USA) was placed for film dosimetry evaluation. A overview of the experimentail setup can be found in the supplemetary materials (Supplementary Figure S1)

### 2.2 Target definition

For the target definition step (Fig. 1), a PET acquisition was performed on the Biographic mCT scanner (Siemens Healthineers, Knoxville, USA) using the clinical PET acquisition protocol with list-mode data, with and without motion of the insert to establish a static reference. Motion correction to mid-position was performed using the PET Maximum-Likelihood Motion and Activity (MLMA) framework [15]. The motion estimation step incorporated two innovations: i) Motion compression: motion was modelled to reduce the number of parameters needed to solve for the Cubic B-spline approach used. ii) Motion smoothness: motion was assumed spatially smooth, by including a spatial regularization penalizing the spatial curvature.

Next, a motion-corrected reconstruction was performed by incorporating the 3D motion vector fields determined by the motion estimation step. The reconstruction method applied the motion vector fields within the ordered subset expectation maximisation (OSEM) loop of the iterative reconstruction. The framework was configured to use the clinical corrections for normalization, scatters and randoms as estimated by the scanner console.

Motion-corrected and motion-blurred PET scans were used for target definition via SUV_max_ thresholding. Threshold values were empirically chosen, resulting in a 50% threshold for the boost volume and a 30% threshold for the GTV volume in phantom experiments. Both GTV and GTV_boost_ were expanded by a 3 mm margin to form a Planning Target Volume (PTV). Based on these volumes, a 5 × 5 Gy + 1 Gy integrated-boost Intensity-Modulated Radiation Therapy (IMRT) plan was created in Monaco (Elekta AB, Stockholm, Sweden).

### 2.3 Treatment delivery

The treatment delivery step (Fig. 1) was performed on a Unity MR-linac (Elekta AB, Stockholm, Sweden). An MRI of the phantom was acquired, and the RT-plans were adapted by rigid registration of the planning PET/CT to the online MRI, within Monaco (Elekta AB, Stockholm, Sweden).

Three plans (static, motion-blurred and motion-corrected) were delivered to the phantom. During static RT-plan delivery, the insert remained static to serve as a reference. During delivery of the motion-corrected and motion-blurred PET based RT-plans, the insert was in motion. One fraction of each plan was delivered with and without intra-fraction motion mitigation using gated delivery or Multi Leaf Collimator (MLC)-tracking (Table 1).

**Table 1:** Overview of the motion behavior of the phantom in each experiment. Rows indicate the treatment plans used, and columns indicate the delivery methods. Here −- indicates that the phantom had no motion both during PET acquisition and treatment delivery and ++ the phantom had motion during PET acquisition and treatment delivery.

	PET Static	Motion Blur	Motion corrected
Static delivery	−-	++	++
Gated		++	++
MLC tracking		++	++

For gated delivery, the comprehensive motion management (CMM) software (Elekta AB, Stockholm, Sweden) was used. Motion monitoring of the GTV was performed using two dimensional (2D) interleaved sagittal and coronal cine-images with balanced gradient echo sequence at 2 Hz per plane, resulting in 4 Hz temporal resolution. The PTV served as the gating envelope. During delivery, the beam was automatically gated if overlap between the GTV and gating envelope (PTV) dropped below 95%.

A real-time loop between the MRI system and the MLC controller was implemented for MLC tracking. Acquisition of 2D sagittal cine MRI frames was performed at 4 Hz and the data were streamed to in‑house developed tracking software [16]. The target position was estimated using normalized cross-correlation, and used to calculate shifted MLC apertures, which were then sent asynchronously to the MLC controller at a rate of 25 Hz. The MLC controller software was modified to accept such incremental aperture updates. A linear ridge regression prediction filter was implemented to compensate for the end-to-end latency of 300 ms [17].

For the film dosimetry, EBT3 film was used. Twenty-four hours after irradiation, the films were digitized using an EPSON 1000XL flatbed scanner (ESPON, Luxenbourg, Luxemburg) at a resolution of 150 dots per inch. The films were processed using in-house–developed software performing triple-channel dose analysis with lateral corrections. Point registration was based on three reference points in the corners of each film. Correspondence between the static reference, the motion-blurred and motion-correct film was assessed through dose differences and 3%/3 mm global gamma pass-rates analysis.

### 2.4 Patient simulation

For the simulation, data from a patient scanned for metastatic lung lesions with ^18^F-FDG was included. Informed consent was waived by the Institutional Review Board. List-mode data was used to reconstruct standard and motion-corrected PET-images using the PET-MLMA framework. This resulted in a motion-blurred PET-image and a motion-corrected PET-image.

The image contained four lesions with increased FDG uptake in the right lung, of which one was located near the liver-lung interface. This lesion was delineated using a 40% SUV_max_ thresholding method [1], and a 2 mm PTV margin was added. Based on these delineations, an 8 × 7.5 Gy IMRT plan was created in Monaco for each image set, resulting in two plans: one based on the motion-blurred image and one on the motion-corrected image.

One fraction of each plan was delivered on the MR-linac. Dosimetry was assessed with the Delta4 Quality assurance (QA) system [18]. The Delta4 Phantom+ (Scandidos AB, Uppsala, Sweden) was placed on the QUASAR moving platform (IBA Dosimetry GmbH, Schwarzenbruck, Germany). The center of the Delta4 was positioned at isocenter in superior-inferior (SI) direction. In the left–right (LR) direction the phantom was placed with an offset of 6 cm to ensure that the high dose areas of the plan coincided with the high-density diode grid at the center of the Delta4 Phantom. Target motion was simulated using a 1D waveform extracted from the motion fields estimated by PET-MLMA (cranio-caudal liver–lung motion). All of the PET-based plans were recalculated on the Delta4 prior to delivery. The plans were delivered using a static delivery, gating and MLC-tracking. Position estimation for gating and MLC tracking was based on the phantom signal, as MR imaging of the Delta4 phantom was not possible. The gating window was selected such that beam delivery occurred when 95% coverage of the PTV was achieved.

Dose evaluation included dose differences and 2%/2 mm global gamma pass-rates analysis, with the static delivery of the motion-corrected RT plan where the phantom was kept static during delivery serving as the reference.

## 3 Results

For the phantom experiment the target volumes were determined for the static reference PET image, the motion-blurred PET image and the motion-corrected PET image.

**Fig. 2:**
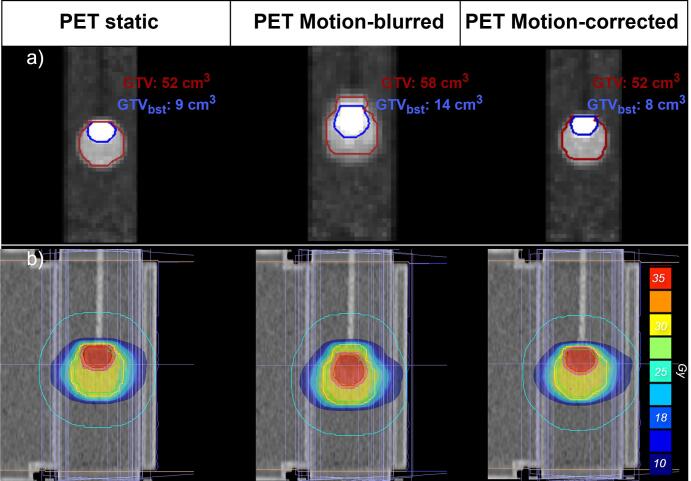
Delineation and RT-plan of the static PET, motion-blurred PET and the motion-corrected PET. a) the target delineations constructed on the PET scans with a threshold of 50% SUV_max_ for the GTV_boost_ and 30% SUV_max_ for the GTV. b) The corresponding 5 x 5 Gy + 1 Gy IMRT plans.

The GTV measured 52 cm^3^ in the static reference PET, 58 cm^3^ in the motion-blurred PET, and 52 cm^3^ in the motion-corrected PET. A similar trend was observed for the GTV_boost_: 9 cm^3^ for the static reference, 14 cm^3^ for the motion-blurred, and 8 cm^3^ for the motion-corrected PET (Fig. 2a). Based on these delineations, RT plans were generated in which the GTV received 5 Gy and the GTV_boost_ received 6 Gy per fraction (Fig. 2b).

**Fig. 3:**
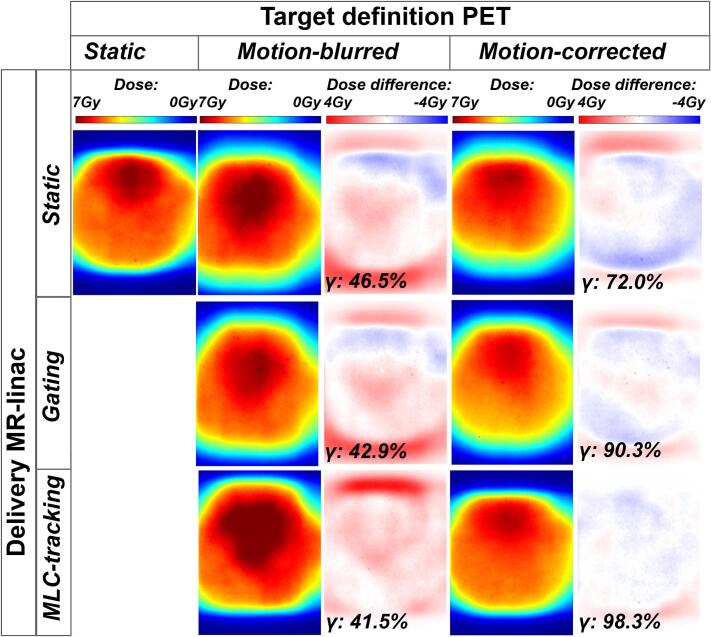
Film dosimetry results for motion-blurred and motion-corrected plans, delivered using static delivery, gated and MLC tracking. The static reference (upper left corner) corresponds to the static treatment plan delivered without phantom motion. The absolute dose, the dose difference compared to the static dose results and pass rate for a 3%/3 mm gamma analyses are shown.

The 2D dose maps for the static reference (i.e., static delivery of the static plan without phantom motion), as well as for the motion-blurred and motion-corrected plans delivered with and without gating or MLC-tracking, are shown in Fig. 3. The motion-blurred RT plans exhibited substantial dose discrepancies under static delivery, gated delivery and MLC tracking, with gamma pass rates of 46.5%, 42.9% and 41.5%**,** respectively. When PET-based target definition was motion-corrected, the dose deviation relative to the static reference decreased. The gamma pass rate improved to 72% under static delivery. The combination of motion correction in both the target definition PET and delivery further minimized dose differences, yielding a gamma pass rate of 90.3% for gated delivery and 98.3% MLC tracked delivery**.**

**Fig. 4:**
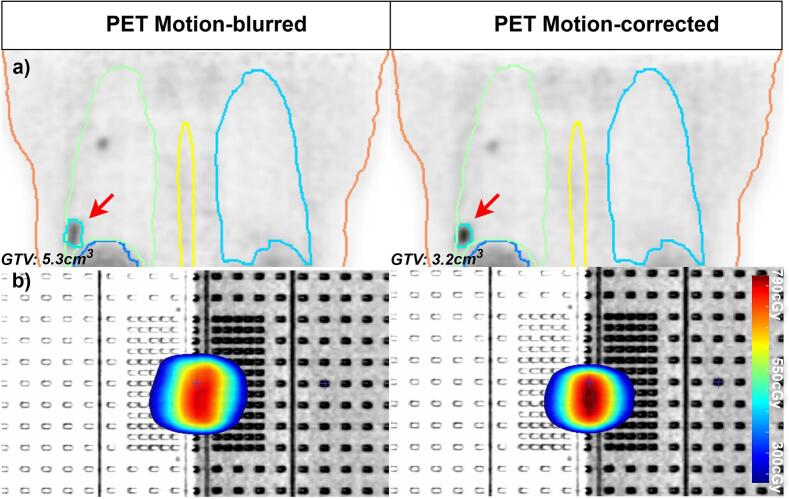
Delineation and RT-plan of motion-blurred PET and the motion-corrected PET of the patient simulation. a) target delineations constructed on the PET scans with a threshold of 40% SUV_max_ for the GTV. b) The corresponding 8 x 7.5 Gy IMRT plans calculated on the Muliti-dioded QA phantom.

For the patient simulation the target volume size was determined for both the motion-blurred PET and the motion-corrected PET. The GTV from the motion-blurred PET measured 5.3 cm^3^ which was larger than the 3.2 cm^3^ GTV from the motion-corrected PET (Fig. 4a). The resulting plans created from these delineations were calculated on a cylindrical multi-diode phantom (Fig. 4b).

**Fig. 5:**
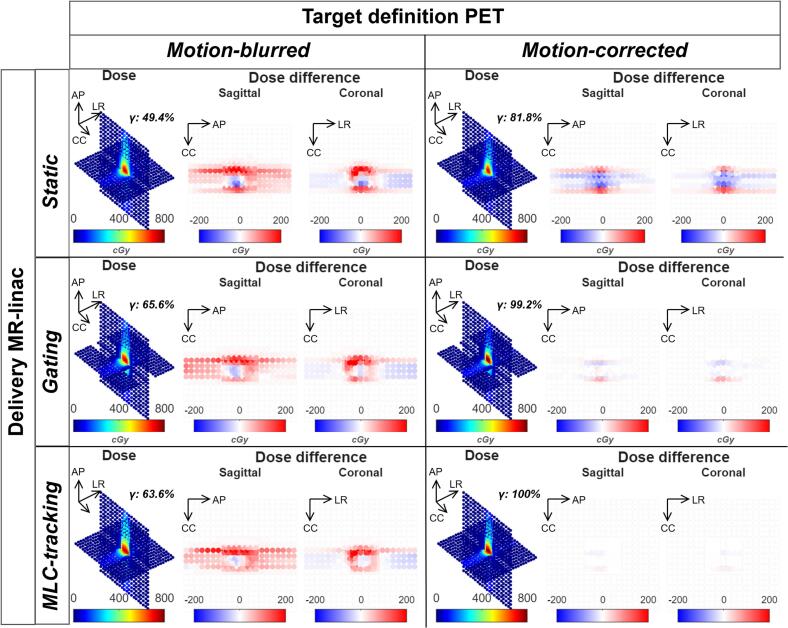
Plan-QA dose results for PET-based static, motion-blurred, and motion-corrected plans, with and without gating and MLC tracking. Absolute dose, dose difference, and 2%/2 mm gamma analyses are shown, using a motion-corrected treatment plan delivered statically and without phantom motion as the reference.

The sagittal and coronal dose maps for the motion-blurred and motion-corrected plans, with static beam, gating or MLC tracking delivery, are shown in Fig. 5. Dose difference maps, calculated relative to the reference plan (the motion-corrected plan delivered statically without phantom motion), revealed large discrepancies for deliveries performed without gating or MLC-Tracking. The motion-blurred plan with MLC-tracking and gating also revealed a maximum dose difference of 2 Gy. Gamma pass rates for motion-blurred plans were 49.4% with static delivery, 65.6% with gated delivery and 63.6% with MLC tracking. In comparison, motion-corrected plans achieved a pass rate of 81.8% with static delivery, 99.2% with gated delivery and 100% with MLC tracking.

## 4 Discussion

In this study, we demonstrated the technical feasibility of a motion robust PET/CT-to-MR-linac pipeline. Motion correction of PET images was achieved using the PET-MLMA framework, while motion-resolved delivery was facilitated by the MR-guided MLC tracking and gating workflow on the MR-linac. Together, these components enabled precise PET-guided radiotherapy.

The target volume derived from the motion-blurred PET image was larger than the static reference, particularly for the boost volume, which nearly doubled due to motion blurring, a well-known effect [6]. This indicates that smaller volumes are more affected by motion during PET acquisition. Such discrepancies can lead to systematic errors in the dose distribution. However, using motion-corrected PET for target definition mitigated these errors and achieved a target volume with sub-voxel accuracy compared to the static reference ground truth, indicating effective motion correction by PET-MLMA. These findings align with studies reporting reduced target volume when applying clinical available motion-correction methods [19], [20]. However, such methods solely correct for periodic motion by means of phase binning. In contrast, PET-MLMA can resolve both periodic and irregular motion by estimating the time-series of deformations at high temporal resolution (2 Hz) for the whole acquisition and the motion-corrected activity image.

Film dosimetry confirmed that the motion-blurred PET-based plan, when delivered statically, suffered from substantial dose discrepancy, reflected in large dose differences and low gamma pass rates. Although the MLC-tracking and gated delivery reduced the effect of motion blurring in the 2D dose distribution, it did not improve the dose difference or gamma pass rate. This is caused by the larger target volume of the motion-blurred PET image, which led to a high dose differences with respect to static plans in some areas. This may increase the risk of unforeseen toxicity. Notably, gamma pass rates were lower for motion-managed delivery methods compared with static delivery. This observation may be explained by motion-induced blurring, which averages out localized overdosing in the non-motion-managed case. In contrast, the plan based on the motion-corrected PET improved the gamma pass rate and reduced dose differences, especially when combined with MLC-tracked and gated delivery. These results highlight the potential of the proposed PET/CT-to-MR-linac pipeline to deliver precise, motion-resolved PET-guided radiotherapy.

Gated delivery yielded a lower gamma pass rate compared to MLC-tracked delivery. This could be attributed to the 95% overlap threshold, which allowed some blurring of dose delivery and consequently resulted in a lower gamma pass rate. Moreover, gated delivery is less efficient, as the beam is turned off when the target moves outside the gating envelope. In contrast, MLC-tracked delivery enables continuous beam-on time, leading to shorter treatment durations. Nevertheless, a major advantage of gating is its clinical availability [21]. Notably, the biggest impact on the dose accuracy originated from motion correction during target definition. This suggests that in cases of respiratory motion without drift, PET motion correction alone may already provide substantial benefit.

The patient simulation further demonstrated that the proposed pipeline can accommodate treatment of patients with small lesions, despite patient motion. This underscores the potential of the PET/CT-to-MRL pipeline in treating oligometastatic disease, which has been proposed as a promising application for PET-guided radiotherapy [22]. While no strict cut-off exists for the number of metastases eligible for treatment, patients with fewer than five lesions are typically considered in oligometastatic NSCLC [23]. The precision of this pipeline could potentially extend this limit, enabling safe treatment of additional lesions.

Accurate registration between PET/CT and MRI is a critical component of the PET/CT‑to‑MR‑linac workflow, as misalignment caused by differing motion states can lead to dose errors. In this study, such errors were avoided by acquiring CT and MRI in a static phantom. In clinical practice, continuous breathing complicates registration. Potential solutions include breath‑hold imaging, although this has practical limitations, or motion‑corrected MRI. Frameworks such as MR‑MOTUS [24], which are based on principles similar to PET‑MLMA, may enable consistent motion correction across PET/CT and MRI. Alternatively, clinically available mid‑position CT and MR images can be used for registration.

Precise PET‑guided radiotherapy is particularly important for dose painting, where misalignment can cause substantial dose errors in high‑dose regions and contribute to unintended toxicity [2], [24]. While motion effects may be limited in locally advanced NSCLC [25], the proposed pipeline could expand PET‑guided boosting to anatomically complex and motion‑affected sites such as the pancreas, liver and small lung lesions near the diaphragm.

This study has limitations. Only one‑dimensional respiratory motion was considered, whereas more complex motion patterns may yield different results. However, the employed motion‑management strategies are generalizable to 3D non‑rigid motion. Additionally, ground truth target volumes were unavailable in the patient simulation, although the observed reduction in target volume after motion correction indicates a measurable effect.

Overall, a motion-robust PET/CT-to-MR-linac pipeline employing motion-corrected PET imaging and MR-guided MLC tracking or gating is technically feasible and enhances dose precision. Here motion correction in the target definition had the greatest impact. This makes it suitable for PET-guided radiotherapy cases that rely on high dose delivery to smaller volumes, like dose painting, PET based adaptive radiotherapy or PET-guided boost. The ability of delivering precise motion-resolved PET-guided radiotherapy has the potential to improve outcomes in these treatments. The pipeline could already be implemented clinically when using clinical available motion mitigation methods. However, the clinical effectiveness of the pipeline in PET-guided radiotherapy application warrants further investigations.

## Declaration of generative AI in scientific writing

During the preparation of this work the author(s) used open ai chatgpt in order to review the text on grammar and spelling. After using this tool/service, the author(s) reviewed and edited the content as needed and take(s) full responsibility for the content of the publication

## CRediT authorship contribution statement

**Albert J. Everard:** Writing – original draft, Visualization, Validation, Methodology, Investigation, Formal analysis, Data curation, Conceptualization. **Rodrigo José Santo:** Writing – review & editing, Software, Investigation. **Pim T.S. Borman:** Writing – review & editing, Software, Investigation. **Prescilla Uijtewaal:** Writing – review & editing, Methodology, Investigation. **Martin F. Fast:** Writing – review & editing, Conceptualization. **Hugo W.A.M. de Jong:** Writing – review & editing, Supervision. **Cornelis A.T van den Berg:** Writing – review & editing, Supervision. **Bas W. Raaymakers:** Writing – review & editing, Conceptualization. **Casper Beijst:** Writing – review & editing, Supervision, Funding acquisition, Conceptualization.

## Declaration of competing interest

The authors declare that they have no known competing financial interests or personal relationships that could have appeared to influence the work reported in this paper.
