# Supplementary material for: Motion mitigation in positron-emission tomography guided radiotherapy delivered on a magnetic resonance imaging-linear accelerator
Source: Phys Imaging Radiat Oncol. 2026 Apr 17;39:100970. doi: 10.1016/j.phro.2026.100970 (PMC13127619; doi:10.1016/j.phro.2026.100970)
Supplement: Supplementary Data 1 [file mmc1.pdf]

Appendix A:

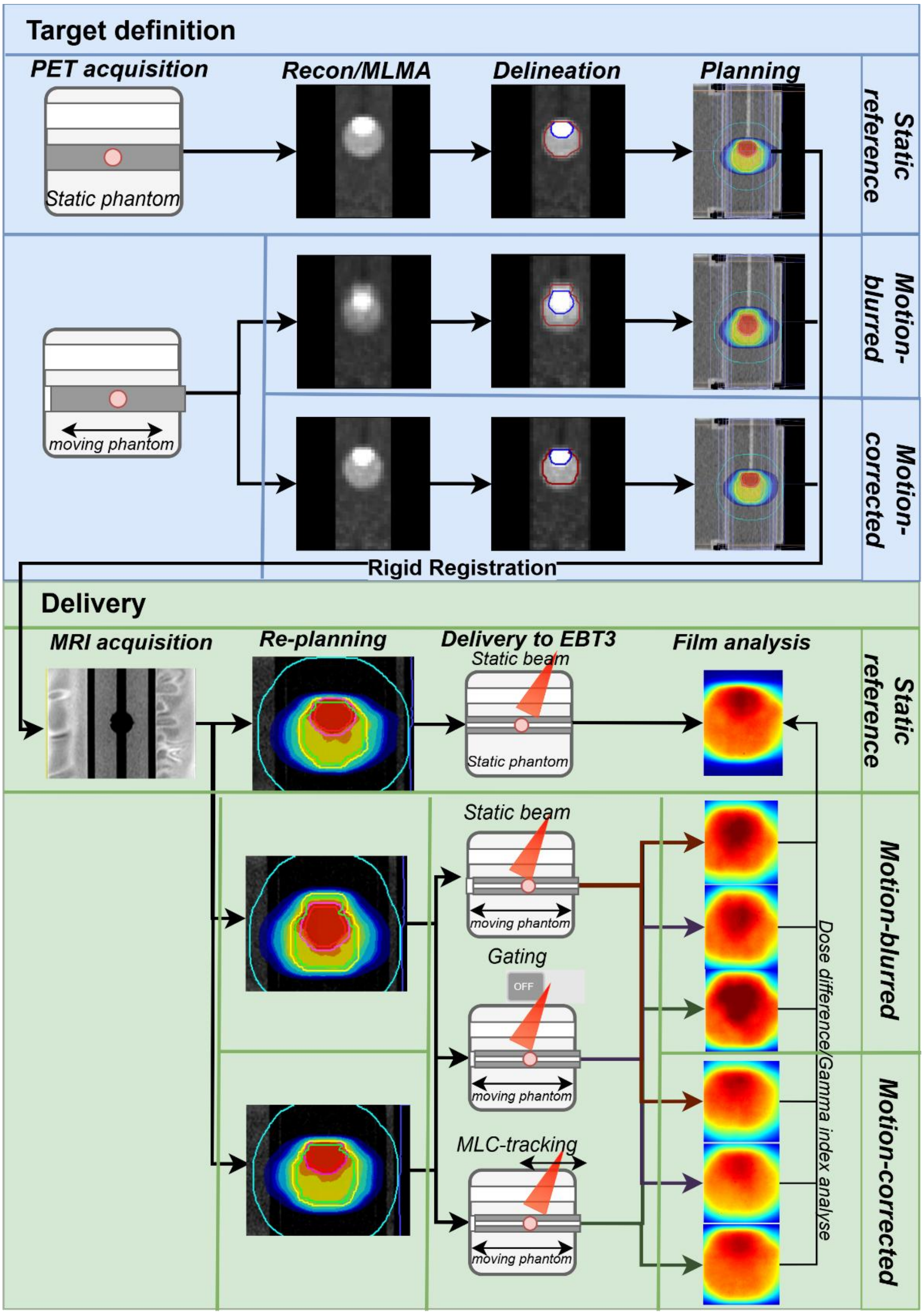

Fig. s1: Overview of the experimental set-up. The target definition steps are indicated in blue and include PET acquisition of raw list-mode data, reconstruction and motion correction, target delineation, and radiotherapy planning. The treatment delivery steps, shown in green, are performed on the MR-linac and consist of MR imaging, adaptation of the radiotherapy plans, and treatment delivery with or without gating and MLC tracking, followed by film analysis.
